# Spent brewer’s yeast as a selective biosorbent for metal recovery from polymetallic waste streams

**DOI:** 10.3389/fbioe.2024.1345112

**Published:** 2024-03-12

**Authors:** Anna Sieber, Leon Robert Jelic, Klemens Kremser, Georg M. Guebitz

**Affiliations:** ^1^ K1-MET GmbH, Linz, Austria; ^2^ Department of Agrobiotechnology, IFA-Tulln, Institute of Environmental Biotechnology, University of Natural Resources and Life Sciences Vienna BOKU, Tulln an der Donau, Austria; ^3^ Austrian Centre of Industrial Biotechnology, Tulln an der Donau, Austria

**Keywords:** biosorption, brewer’s yeast, low-cost biosorbent, selective metal recovery, printed circuit board leachate solutions

## Abstract

While the amount of electronic waste is increasing worldwide, the heterogeneity of electronic scrap makes the recycling very complicated. Hydrometallurgical methods are currently applied in e-waste recycling which tend to generate complex polymetallic solutions due to dissolution of all metal components. Although biosorption has previously been described as a viable option for metal recovery and removal from low-concentration or single-metal solutions, information about the application of selective metal biosorption from polymetallic solutions is missing. In this study, an environmentally friendly and selective biosorption approach, based on the pH-dependency of metal sorption processes is presented using spent brewer’s yeast to efficiently recover metals like aluminum, copper, zinc and nickel out of polymetallic solutions. Therefore, a design of experiment (DoE) approach was used to identify the effects of pH, metal, and biomass concentration, and optimize the biosorption efficiency for each individual metal. After process optimization with single-metal solutions, biosorption experiments with lyophilized waste yeast biomass were performed with synthetic polymetallic solutions where over 50% of aluminum at pH 3.5, over 40% of copper at pH 5.0 and over 70% of zinc at pH 7.5 could be removed. Moreover, more than 50% of copper at pH 3.5 and over 90% of zinc at pH 7.5 were recovered from a real polymetallic waste stream after leaching of printed-circuit boards. The reusability of yeast biomass was confirmed in five consecutive biosorption steps with little loss in metal recovery abilities. This proves that spent brewer’s yeast can be sustainably used to selectively recover metals from polymetallic waste streams different to previously reported studies.

## Graphical Abstract

**Figure:**
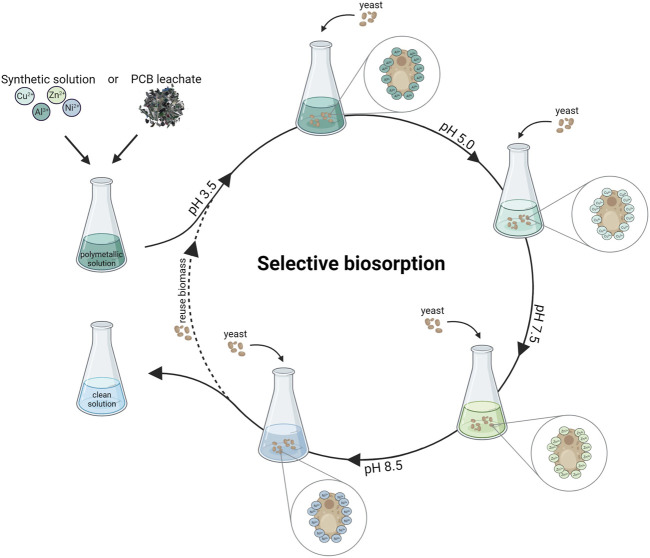
The graphical abstract was created with BioRender.com.

## 1 Introduction

In 2019, 53.6 Mt of e-waste was generated worldwide, and it is predicted to exceed 74 Mt annually in 2030. While this rapid increase in the amount of e-waste is an environmental threat, it also provides a promising resource for valuable and critical materials (Srivastav et al., 2023). Recovering these components from various e-waste sources would reduce the waste deposition problem and help solve supply issues of certain materials (Zulkernain et al., 2023). Bioleaching has emerged as a promising method for metal recovery from e-waste; solubilizing valuable metals from different waste substrates. Among the e-waste streams, printed circuit boards (PCBs) are the most heterogenous, containing heavy metals as well as precious metals and non-metal elements (Baniasadi et al., 2019). Successful PCB bioleaching has been performed in various studies using different microorganisms (Gu et al., 2017; Zulkernain et al., 2023), but the selective recovery of the individual metals from the polymetallic leachate solutions remains challenging. Here, chemical precipitation is still one of the most widely and industrially applied processes. Kremser et al. (2022a) presented a combined approach where after bioleaching of basic oxygen furnace slag, metals like aluminum, manganese, chromium and vanadium could be selectively recovered by the addition of sodium hydroxide. Nevertheless, this process is not suitable for large amounts of waste solutions since it produces a high amount of contaminated sludge (Costa et al., 2021). Solvent extraction is another method for the recovery of metals using an organic solvent phase. However, when it comes to multicomponent solutions containing elements with similar chemical and physical characteristics, solvent extraction faces some challenges (Costa et al., 2020). Additionally, ion exchange is an effective technology for metal recovery, but the resins are rather costly, especially when compared to cheap biosorbents which can sometimes even be obtained from waste streams (Volesky, 2001; Yaashikaa et al., 2021).

Promising results have been obtained by a variety of studies using biosorption as a strategy for metal removal. Biosorbents such as bacteria (Rizvi et al., 2020), algae (Bilal et al., 2018) and natural materials like chitosan (Abhinaya et al., 2021) or clay (Uddin, 2017) have been tested for their metal removal capacities. Additionally, biochar has attracted notable attention in recent years because of its good sorption ability for heavy metals. Despite the advantages of using biochar for metal adsorption, the separation of powdered biochar from wastewater might be challenging due to the small particle size and lower density (Son et al., 2018). An alternative is fungal biomass which offers various advantages such as a high cell wall content with several functional groups available for metal binding. *Saccharomyces cerevisiae* can be obtained as the second major by-product from the brewing industry where the average amount of residual yeast from a larger fermentation is approximately 2.7 kg per m^3^ of the final volume of beer (Rachwał et al., 2020). Hence, some of the obvious advantages of using brewer’s yeast as biosorbent are the availability of large quantities of waste biomass, its low cost and eco-friendliness (Costa et al., 2021). Besides, the chemical composition of *Saccharomyces cerevisiae*, including functional groups on the cell surface, is well described in literature, facilitating the discovery of structural changes upon metal binding and the understanding of the mechanisms involved (Zinicovscaia et al., 2023). Nevertheless, there is limited published work exploring the potential of inactive residual *S. cerevisiae* from the brewing industry (Gonçalves et al., 2023).

High metal recovery rates were achieved in multiple biosorption studies (Yaashikaa et al., 2021); however, these studies mostly focus on removing single-metal ions. Fewer studies were conducted with real wastewater considering the influence of multi-metal ions (Torres, 2020; Priyadarshanee and Das, 2021). Futalan et al. for example, showed a 1.06–1.44 fold and 1.32–1.81 fold decrease in adsorption capacity for Cu and Ni respectively, in a mixed solution when compared to a single-metal solution, using immobilized chitosan as a biosorbent (Futalan et al., 2011). In a study by Bouhamed and co-workers, activated carbon from datestones was used to remove Cu^2+^, Ni^2+^, and Zn^2+^ from real wastewater reporting an adsorption affinity order of Cu^2+^ > Ni^2+^ > Zn^2+^ at pH 5.5 (Bouhamed et al., 2016). Furthermore, Kulkarni and co-workers used industrial waste sludge containing *Saccharomyces carlsbergensis* and reported competitive biosorption of Ni^2+^ and Cd^2+^ and therefore a decrease in metal uptake with increasing co-ion concentration (Kulkarni et al., 2019). Table 1 summarizes the adsorption potential of *S. cerevisiae* and other fungi from polymetallic solutions. In addition to the competitive binding behavior of the metal ions, the low selectivity of the biosorption process in general presents a disadvantage of this technology (Yu et al., 2020). Some studies showed enhanced metal uptake after treating the yeast biomass with organic solvents (Mapolelo and Torto, 2004; Yaashikaa et al., 2021) or alkaline solutions (Farhan and Khadom, 2015; Mirmahdi et al., 2022) while additionally increasing the selectivity by changing the solution pH (Kulkarni et al., 2019) or by displaying metal-binding peptides on the surface of the biosorbent (Li et al., 2019).

**TABLE 1:** Biosorption capacity of different industrial waste biosorbents from polymetallic solutions.

Biosorbent	Biomass [g l^−1^]	Metals	Metal conc. [mg l^−1^]	pH	Contact time [h]	Recovery [mg g^−1^]	References
Pulverized biomass of *Mucors sp*. NRCC6	1	Pb^2+^	100	5.5	0.5	15.0	El-Gendy et al. (2023)
		Ni^2+^				9.0	
		Zn^2+^				5.0	
		Mn^2+^				2.84	
*S. cerevisiae* ethanol pre-treated	1	Cu^2+^	25	5	3	7.35	Göksungur et al. (2005)
		Cd^2+^				2.93	
		Pb^2+^				7.88	
*S. cerevisiae* ethanol pre-treated	1	Cu^2+^	25	4	3	5.38	Göksungur et al. (2005)
		Cd^2+^				2.80	
		Pb^2+^				6.48	
Brewery sludge	4	Ni^2+^	50	6.0	2	2.92	Kulkarni et al. (2019)
		Cd^2+^				7.54	
*S. cerevisiae* biomass	0.75	Fe^3+^	50	2	24	12.1	Goyal et al. (2003)
		Cr^4+^					
*S. cerevisiae* biomass	0.75	Cr^4+^	150	2	24	27.5	Goyal et al. (2003)
		Fe^3+^	50				
*S. cerevisiae* biomass	10	Zn^2+^	10	2–6	0.5	1.2	Zinicovscaia et al. (2020)
		Ni^2+^	2			0.13	
		Cu^2+^	2			0.11	
*S. cerevisiae* lyophilized	10	**Al** ^**3+**^	100	3.5	0.5	**5.32**	Present study
		Cu^2+^				1.50	
		Zn^2+^				0.38	
		Ni^2+^				0.27	
*S. cerevisiae* lyophilized	10	**Cu** ^**2+**^	85	5.0	0.5	**4.24**	Present study
		Zn^2+^	96			1.46	
		Ni^2+^	97			1.13	
*S. cerevisiae* lyophilized	10	Cu^2+^	43	7.5	0.5	1.54	Present study
		**Zn** ^**2+**^	82			**7.48**	
		Ni^2+^	86			2.23	
*S. cerevisiae* lyophilized	10	Cu^2+^	27	8.5	0.5	0.54	Present study
		Zn^2+^	7			**0.78**	
		**Ni** ^**2+**^	64				

Interestingly, all the aforementioned studies describe the adsorption behavior of different biosorbents from mixed metal solutions at only one distinct pH value. In this study, different chemical and physical pre-treatment methods such as incubation with green solvents or at various temperatures were tested to optimize the yeast surface properties and hence improve selective metal binding. For the first time, a stepwise biosorption process is proposed where the economically important metals aluminum, copper, zinc, and nickel are selectively recovered out of polymetallic solutions onto spent brewer’s yeast surface with changing the solution pH. This provides an efficient and sustainable process for recovering multiple metals from real liquid waste streams. After desorption of the metal ions, the yeast biomass could be recycled and used up to 5 times making the process even more cost-effective.

## 2 Materials and methods

Chemicals of analytical grade were purchased from Sigma-Aldrich (Sigma-Aldrich, Austria) unless otherwise specified. The pH-value of different metal solutions was measured with a Mettler Toledo S220 pH-meter with a combined glass electrode (Mettler-Toledo GmbH, Austria).

### 2.1 Determination of metal concentrations

Metal concentrations in single-metal solutions were determined by using commercially available photometric cuvette tests LCK329, LCK360 or LCK337 (Hach Lange, Austria) for copper, zinc, or nickel respectively and were measured on a DR3900 spectrophotometer (Hach Lange, Austria). For the analysis of aluminum contents, a spectrophotometric assay based on the complex formation of metals with xylenol orange was set up. Briefly, 20 µl of sample was added to 1860 µl of potassium hydrogen phthalate buffer (0.05 M, pH 3.0). After addition of 120 µl of xylenol orange (1.58 * 10^−3^ M), samples were incubated at 70°C for 8 min in an Eppendorf^®^ Thermomixer comfort (Eppendorf, Austria) to ensure complete color reaction. Afterwards, samples were cooled to approximately 22°C and the absorbance of 250 µl of each sample was measured with a TECAN Infinite 200 PRO M Plex Microplate Reader (Tecan, Switzerland) at 550 nm with 25 flashes per sample and a bandwidth of 9 nm. First, a calibration curve with samples of known aluminum concentrations ranging from 0 mg l^−1^ to 1.4 mg l^−1^ was created and this was subsequently used for the calculation of the aluminum content of the unknown samples.

For the determination of the metal concentration in polymetallic solutions, samples were measured with inductively coupled plasma mass spectrometry (ICP-MS) as described in a previous study (Kremser et al., 2021). Briefly, samples were diluted with ultrapure water by a factor of 100 before analysis. A solution of Sc (400 μg l^−1^) was used as an internal standard to suppress possible matrix effects.

Biosorption experiments were performed using either 50 ml or 100 ml of metal solutions in Erlenmeyer flasks on a KOMET Variomag Poly stirring plate (Thermo Fisher Scientific, United States). All centrifugation steps were performed with an Eppendorf^®^ Centrifuge 5920 R (Eppendorf, Austria) for 10 min at 3,153 x g unless stated otherwise.

### 2.2 Yeast biomass

Around 20 l of spent brewer’s yeast (*S. cerevisiae*) were obtained from a local brewing company at the end of beer brewing process and centrifuged in 1-liter aliquots to separate yeast biomass and liquid brewing residues using a Sorvall Lynx6000 superspeed centrifuge (Thermo Fisher Scientific, United States) at 17,568 x g for 20 min. Cell pellets obtained after centrifugation were washed by resuspension in 1 liter deionized water followed by centrifugation. This washing step was repeated three times to remove remaining alcohol and sugars from the brewing process. After centrifugation, the yeast pellets were frozen at −20°C. Frozen yeast biomass was lyophilized using a CHRIST^®^ Alpha 1-4 LDplus freeze dryer (CHRIST, Germany), ground to a fine powder using a kitchen blender and stored in a benchtop desiccator to protect it from moisture. Sieve analysis was used to obtain the particle size distribution using a stacked sieve tower with mesh sizes ranging from 2 mm to 0.1 mm.

### 2.3 Pre-treatments of yeast biomass

#### 2.3.1 Physical pre-treatment

In addition to lyophilization, spent brewer’s yeast was oven-dried at 70°C until complete dryness in a drying chamber. In the same way, cell dry mass (CDM) of the washed and frozen yeast biomass was evaluated. The dried biomass was ground to a fine powder using a kitchen blender similar to the lyophilization experiments.

For the sonication treatment, 20 g of washed yeast biomass was resuspended in deionized water, placed into a Branson^®^ 5,800 ultrasonic bath (Thermo Fisher Scientific, United States) and sonicated for 15 min at 40 kHz and 45°C. Additionally, autoclaving was performed with 20 g of yeast biomass resuspended in deionized water for 15 min at 121°C using a benchtop autoclave (CertoClav Sterilizer GmbH, Austria). Centrifuged yeast pellets after sonication and autoclaving were stored at −20°C for further experiments.

#### 2.3.2 Chemical pre-treatment

Chemical pre-treatments were performed using lyophilized yeast biomass. For ethanol or sodium hydroxide treatment, 5 g of freeze-dried yeast were resuspended in 62.5 ml of 70% ethanol or 1 M sodium hydroxide solution and stirred for 20 min at 550 rpm in the fume hood. After centrifugation, ethanol treated yeast pellets were washed 3 times and sodium hydroxide treated pellets 10 times with deionized water to remove the remaining ethanol/sodium hydroxide and were stored at −20°C.

2,2,5,5-Tetramethyltetrahydrofuran (TMO), which was synthesized as previously described (Byrne et al., 2017) to >98% purity, and 2-Methyltetrahydrofuran (2-Methyl-THF) (≥99% purity), were tested as green solvents in biomass pre-treatment. Therefore, 6 g of yeast biomass were resuspended in 75 ml of TMO and 2-Methyl-THF each and shaken at 550 rpm over night at 22°C in the fume hood. The biomass was then allowed to settle down and the supernatant containing the solvent was decanted off. After the evaporation of the remaining solvent, yeast pellets were washed 3 times with deionized water and stored at −20°C.

### 2.4 Aqueous metal solutions

Metal stock solutions (1 g l^−1^) were prepared by dissolving 12.33 g of aluminumsulfate octadecahydrate (Al(SO_4_)_3_ · 18 H_2_O), 3.93 g of copper (II)-sulfate pentahydrate (CuSO_4_ · 5 H_2_O), 4.48 g of nickel (II)-sulfate hexahydrate (NiSO_4_ · 6 H_2_O) or 2.75 g of zincsulfate heptahydrate (ZnSO_4_ · 7 H_2_O) in 1 l of deionized water each. For the biosorption tests, metal solutions were diluted to appropriate metal concentrations and the pH was adjusted using 0.5 M sulfuric acid or 0.5 M sodium hydroxide (Honeywell^®^, United States).

### 2.5 Design of experiments (DoE) to establish the experimental conditions

The analytics software MODDE^®^ (Sartorius, Germany) was used to design a set of experiments by varying the factors pH, biomass concentration and metal concentration to test their effect on biosorption efficiencies. The final experimental plan included 18 individual experiments for each metal to be tested. Metal concentrations from 10 to 500 mg l^−1^ and biomass concentrations from 1 to 10 g l^−1^ were tested. To avoid metal precipitation due to increase in sample pH, 3 different pH values for each individual metal were selected with regard to existing literature and evaluated. Aluminum solutions were tested at pH 2.5, 3.0 and 4.0, copper solutions at 2.5, 4.0 and 5.5, zinc solutions at pH 3.0, 5.0 and 7.0 and nickel solutions at pH 3.0, 5.5 and 8.8.

### 2.6 Biosorption experiments

Following DoE experiments, the most promising metal and biomass concentrations of 100 mg l^−1^ and 10 g l^−1^, respectively were applied for further biosorption experiments. For experiments that were performed with chemically or physically pre-treated biomass, the same concentration of biomass in cell dry weight was chosen. All biosorption experiments were performed in duplicate with 100 ml metal solution at 22°C for 60 min, stirring at 300 rpm followed by centrifugation to separate the yeast biomass from the metal solution. Metal concentrations of the supernatants were measured as described above. The amount of metal uptake by the yeast biomass and the biosorption efficiency were calculated using the following equations (Eqs 1, 2):

$$q=\frac{c_{1}-c_{2}}{m}*V$$ (1)

$$E=\frac{\left(c_{1}-c_{2}\right)}{c_{1}}*100$$ (2)

where q [mg g^−1^] is the amount of metal adsorbed per amount of biomass used, m [g]; V [ml] is the volume of the biosorption reaction; E is the efficiency [%]; c_1_ is the initial metal concentration [mg l^−1^] and c_2_ is the metal concentration in the supernatant after the biosorption [mg l^−1^].

### 2.7 pH and temperature optimization experiments

In the optimization experiments, metal concentrations of 100 mg l^−1^ and a biomass concentration of 10 g l^−1^ were used. Contact time and stirring speed were kept constant, while two different pH values for aluminum, copper and zinc (3.0 and 3.5 for aluminum, 5.0 and 5.5 for copper, 7.0 and 7.5 for zinc), one pH value for nickel (8.5) and temperatures of 30°C, 40°C, and 50°C were tested. Optimization experiments were performed in 100 ml Erlenmeyer flasks using 50 ml metal solutions.

### 2.8 Sequential biosorption experiments of individual metals

Sequential biosorption experiments were performed in duplicates with 50 ml single metal solutions containing 100 mg l^−1^ metal in 100 ml Erlenmeyer flasks at pH values of 3.5 for aluminum, 5.0 for copper, 7.5 for zinc and 8.5 for nickel. Following pH adjustment, 10 g l^−1^ freeze-dried yeast was added to the metal solutions and biosorption experiments were performed at 22°C under stirring at 300 rpm. After 30 min, samples were centrifuged at 3,153 x g to separate the yeast biomass and metal solution. The metal concentration in the supernatant was determined, the pH of the metal solution was measured and if necessary, adjusted with 0.5 M H_2_SO_4_ or 0.5 M NaOH. Then, 10 g l^−1^ of fresh biomass were added to the supernatant and the biosorption was continued as described above. This process was repeated until a biosorption efficiency of around 80% was reached.

In addition, bio-desorption experiments for Cu^2+^ and Zn^2+^ containing solutions were carried out. Therefore, the first biosorption step was performed as stated above. After the first centrifugation step, the metal containing supernatant was stored while the yeast cell pellet was resuspended in 25 ml biogenic sulfuric acid (pH 1.2) from a previous study (Kremser et al., 2022b) for bio-desorption of the metal ions and stirred at 300 rpm for 30 min at 22°C. After centrifugation, the yeast pellet was washed once with deionized water and resuspended in metal solution from the first biosorption step. Metal concentrations were measured after each step and the biosorption and bio-desorption process was repeated until a biosorption efficiency of around 80% was reached.

### 2.9 Selective biosorption from polymetallic solutions

Synthetic polymetallic solutions were prepared containing 100 mg l^−1^ of aluminum, copper, nickel and zinc each in 100 ml total volume. After adjusting the pH of the metal solution to 3.5, yeast biomass was added at a concentration of 10 g l^−1^. The biosorption was performed in duplicate for 1 h while stirring at 300 rpm followed by a centrifugation step to separate yeast biomass from the metal solution. The pellet was stored at −20°C, following lyophilization for further sample characterization. The pH of the metal solution was measured, adjusted to 5.0, and 10 g l^−1^ fresh biomass was added for the next biosorption step. This was repeated for pH 7.5 and 8.5. A sample of 1 ml was taken after each biosorption round to determine the metal concentration via ICP-MS.

In a second approach, two different polymetallic bioleachate solutions obtained from printed-circuit boards (PCB) bioleaching with concentrated biogenic sulfuric acid were tested. According to metal concentrations (Table 2), leachates were diluted to reach concentrations found suitable for the individual metals in the DoE screening. Leachate solution 1 was diluted 1:100 whereas the leachate solution 2 was diluted 1:20 in deionized water. Biosorption was performed in duplicates as described for the synthetic solutions.

**TABLE 2:** Metal concentrations of the two different PCB leachate solutions.

Metal ion	Leachate solution 1	Leachate solution 2
mg l^−1^ ± SD		
Aluminum	10.4 ± 4.6	209.8 ± 7.6
Copper	13,943.8 ± 125.5	2,458.7 ± 46.7
Zinc	4,371.9 ± 52.5	1,303.4 ± 15.6
Nickel	184.9 ± 1.1	60.1 ± 1.2

### 2.10 Biomass characterization

To determine the surface characteristics of the lyophilized yeast biomass before and after biosorption, Fourier transformed infrared spectroscopy (FT-IR) measurements were performed on a PerkinElmer^®^ Spectrum 100 spectrometer (PerkinElmer, Austria) between 4,000 and 650 cm^−1^. The samples were measured using 32 scans at a resolution of 2 cm^−1^.

For the visualization of the biomass surface and the adsorbed metal ions, scanning electron microscopy (SEM) coupled with an energy dispersive spectrometer (EDS) was performed on a Hitachi^®^ TM3030 (Hitachi, Japan) at an acceleration voltage of 15 kV for 60 s.

Furthermore, X-ray fluorescence measurements were conducted using a Niton™ XL3t GOLDD + XRF analyzer (Thermo Fisher Scientific, United States) with the default *Soil Mode* settings, a standard filter of 60 and a high and low filter of 20. Samples were measured in triplicates. Values for the corresponding metal peaks in the XRF spectra were averaged and used for statistical analysis using the statistics software SPSS^®^ (version 26) by IBM^®^. Single factor analysis of variance (ANOVA) with multiple repeated measurements was performed to identify significant differences in recovery depending on the four different pH values. Hence, a general linear model with multiple repeated measurements was created by defining the inner subject factors to *pH* as the dependent variable and their number being four. Furthermore, the four pH values were assigned to the subject factor. The H_0_ was defined as no difference between mean peaks at the different pH values tested. The level of significance was 0.05. Bonferroni correction was applied as a *post hoc* comparison. Pairwise comparison of the various combinations of pH values was analyzed and repeated for each metal, respectively.

## 3 Results and discussion

### 3.1 DoE results for single metal biosorption

As a first step, biosorption efficiencies were tested at different metal ion and biomass concentrations ranging from 10 to 500 mg l^−1^ and 1–10 g l^−1^ respectively, and at 3 different pH values for each metal ion. The results of these 18 experiments (Supplementary Figures S1–S4) showed a maximum recovery rate for all the 4 metal ions when using less than 150 mg l^−1^ of metal ion and a biomass concentration higher than 9 g l^−1^. Within these experimental settings it was predicted that the metal recovery of Ni^2+^ at pH 8.0 and Al^3+^ at pH 4.0 is approximately 40% whereas for Cu^2+^ at pH 5.5 and for Zn^2+^ at pH 7.0 metal recovery rates of above 60% can be reached. It was therefore decided to perform all future experiments with a metal ion concentration of 100 mg l^−1^ and 10 g l^−1^ of biomass to reach maximum metal recovery. Due to the large number of binding sites on the yeast surface, high adsorption efficiencies are usually observed during the first few minutes of interaction, whereas equilibrium is reached in approximately 1 h (Farhan and Khadom, 2015; Ojima et al., 2019). Zinicovscaia and co-workers measured the adsorption kinetics of dead yeast biomass in an Ag^+^/Cu^2+^/Ni^2+^/Zn^2+^ system and reported that equilibrium was achieved for all four metals after 45–60 min. These results suggest that electrostatic interactions on the yeast surface are the main driving force of biosorption (Zinicovscaia et al., 2023). Since the biomass used in this study was also dead, the contact time for the biosorption experiments in this study was set to 1 h. In biosorption experiments the pH plays a crucial role in controlling the metal ion speciation in solution and the chemical configuration of the metal-binding functional groups on the biomass surface (Zinicovscaia et al., 2021). Therefore, further pH optimization experiments were conducted.

### 3.2 pH and temperature optimization

To improve the biosorption efficiencies, two additional pH values for Al^3+^, Cu^2+^ and Zn^2+^ and one extra pH value for Ni^2+^ were tested. For Cu^2+^, the biosorption efficiencies at pH 5.0 and 5.5 stayed the same at approximately 42%. For Al^3+^ at pH 3.5 however, the biosorption increased to 60.2% compared to 44.2% at pH 3.0. For Zn^2+^ at pH 7.5 and Ni^2+^ at pH 8.5 a maximum recovery of 30.2% and 19.6% were achieved. As reported in literature, the point of zero charges for *S. cerevisiae* is around pH 4.0 (Stathatou et al., 2022). In a study by De Rossi et al. (2020) a *S. cerevisiae*/alginate composite was used for the adsorption of heavy metals. By encapsulating the yeast biomass, the zero-charge point shifted to a more neutral pH of around 7.0. However, in the present study, no such immobilization method was applied. It was therefore assumed that the pH at which yeast biomass exhibits a charge of zero is in line with the reported value for *S. cerevisiae* itself. Based on this, at pH values below 4.0, protonation of the functional groups on the yeast surface is expected whereas at higher pH values the yeast surface may have an overall negative charge. This could be beneficial for attracting positively charged metal ions, if the biosorption is mainly driven by electrostatic interactions (Stathatou et al., 2022; Zinicovscaia et al., 2023). According to the results of the DoE experiments, pH 3.5 for Al^3+^, 5.0 for Cu^2+^, 7.5 for Zn^2+^ and 8.5 for Ni^2+^ have been proven to be most suitable for removal of the respective metal ion out of single-metal solutions within these experimental conditions.

**FIGURE 1:**
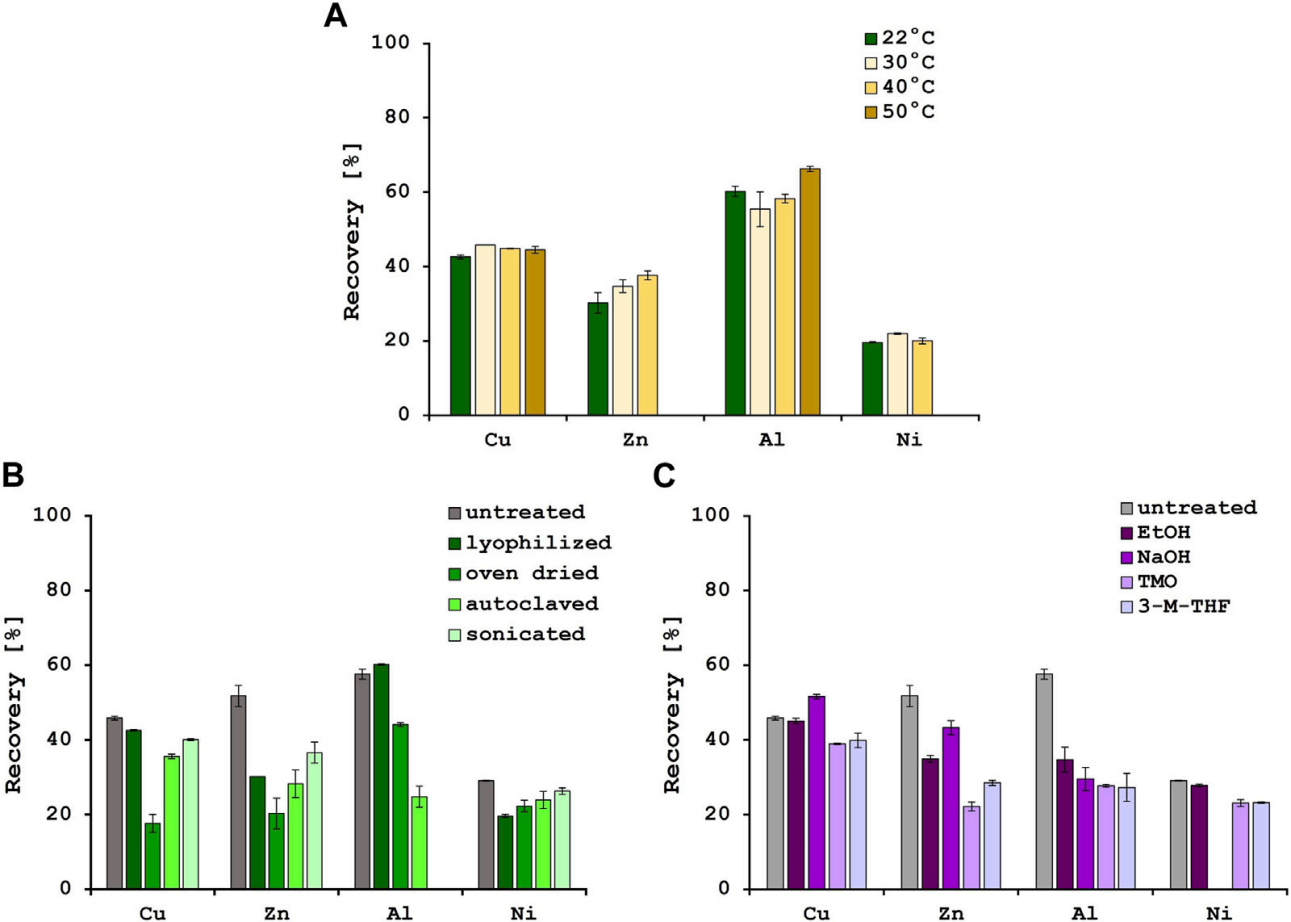
Metal recoveries [%] after biosorption at 4 different temperatures **(A)** and various physical **(B)** and chemical **(C)** pre-treatment methods. Error bars indicate the standard deviation (SD) of two independent experiments (*n* = 2).

Temperature is also reported to have an influence on biosorption efficiencies, although this effect might be more relevant when using living biomass, since the metabolic activity increases when the temperature rises, until an optimum value is reached (Torres, 2020). In this study, biosorption experiments were performed at the following 4 temperatures: 22°C (room temperature), 30°C, 40°C and 50°C (Figure 1A). For Cu^2+^, metal recovery of approximately 45% could be achieved at all 4 temperatures. In addition to this, for Ni^2+^ the recovery stayed the same at approximately 21%. However, for Zn^2+^ recovery an improvement in biosorption efficiencies was observed with rising temperatures, from 30.2% at 22°C to 37.6% at 40°C. At 50°C, there was precipitation visible which was also observed for the Ni^2+^ solution at the highest temperature. The biosorption for Al^3+^ stayed approximately the same with a slight increase from 60.2% to 66.3% at 50°C. Several studies described an increase in biosorption efficiencies for Ni^2+^, Cu^2+^ and Zn^2+^ with decreasing temperatures, indicating an exothermic process (Farhan and Khadom, 2015; Kulkarni et al., 2019; Zinicovscaia et al., 2020). On the other hand, the biosorption of Al^3+^ ions with different biosorbents was often reported as an endothermic process (Costa et al., 2021). In this study, the increase in biosorption with increasing temperature was marginal and only measurable for Zn^2+^ and Al^3+^. It was therefore decided to perform all further experiments at 22°C.

### 3.3 Characterization of yeast biomass

**FIGURE 2:**
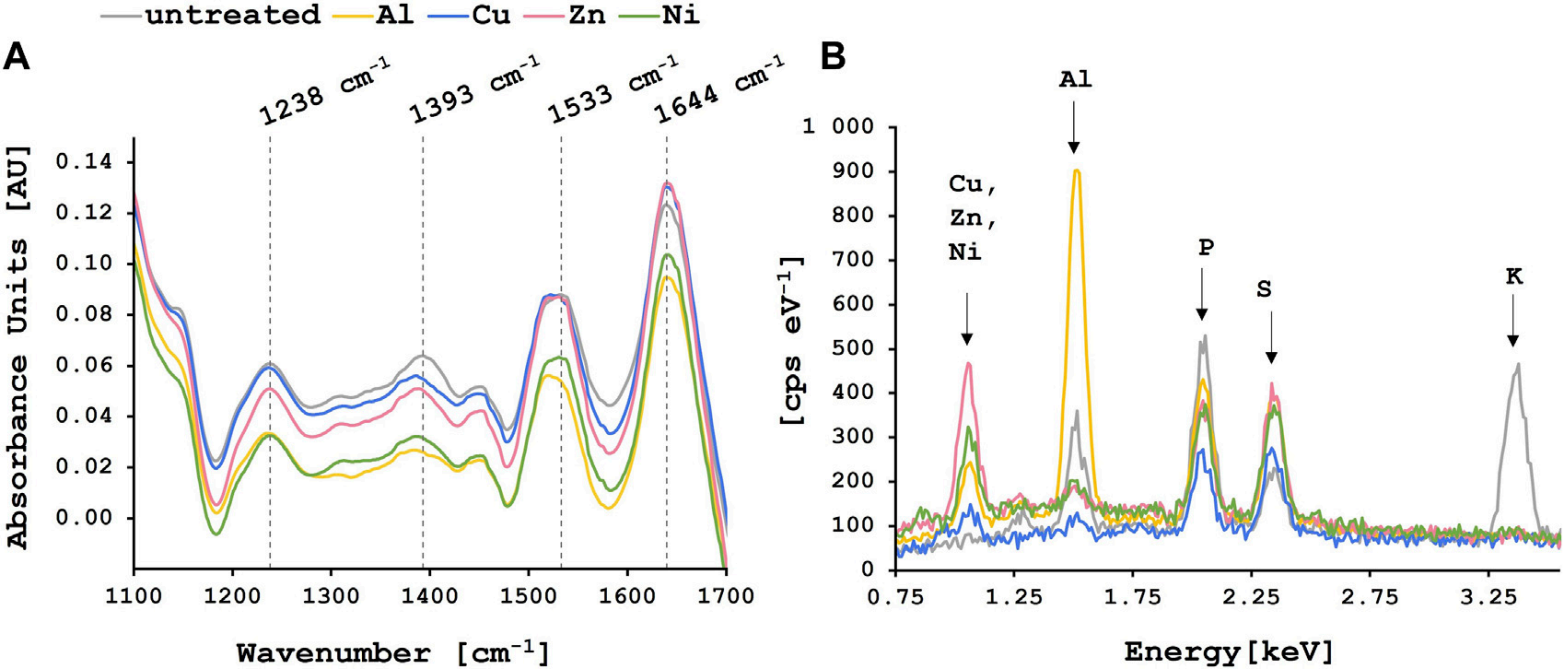
Characterization of lyophilized yeast biomass loaded with or without metal ions. The colored lines correspond to the yeast biomass loaded with the different metal ions whereas the grey line shows the untreated biomass control. **(A)** FT-IR spectra where the dashed lines indicate the characteristic absorbance peaks of lyophilized yeast biomass at the corresponding wavenumbers. **(B)** EDS analysis where the characteristic peaks for the adsorbed elements are labeled.

Following biosorption experiments with single-metal solutions, FT-IR spectra of biosorbent loaded with and without metal ions were recorded. Figure 2A shows the characteristic absorbance peaks of lyophilized brewer’s yeast between 1,100 and 1,700 cm^−1^. The most prominent peaks in this area correspond to the C=O stretching vibration of carboxyl groups and N-H bending of protein amide I band (1,644 cm^−1^), N-H bending and C-N stretching vibration of protein amide II band (1,533 cm^−1^) (De Rossi et al., 2018; Zinicovscaia et al., 2023), C-O stretching vibration of COOH in uronic acid (1,393 cm^−1^) and C-N stretching vibration of amide band II (1,238 cm^−1^) (Dong et al., 2009). After metal adsorption, changes could be observed in the yeast biomass spectra. Especially for the Al^3+^ loaded biomass, where peaks at 1,533 and 1,393 cm^−1^ shifted to approximately 1,520 and 1,385 cm^−1^, respectively. For the Cu^2+^, Zn^2+^ and Ni^2+^ loaded biomass there was a peak shift from 1,533 to 1,527 cm^−1^ observed. These results indicate the involvement of carboxyl and amide functional groups in metal adsorption onto the yeast surface as previously reported by Dong et al. and others (Dong et al., 2009; Machado et al., 2009; Kulkarni et al., 2019).

Surface images of the lyophilized yeast biomass before and after the metal biosorption (Supplementary Figure S5) were taken, including EDS analysis. No morphological changes were observed after metal adsorption. While healthy yeast cells have a round and smooth surface, the yeast cells used in this study had a rough surface and the cell walls looked slightly shriveled. Many authors have described that crushing of the cells leads to the destruction of the cell membranes resulting in an increase in surface area and greater exposure of intracellular components thus increasing the sorbent binding sites (Wang and Chen, 2006; Gonçalves et al., 2023). Additionally, it was reported that the surface of brewer’s yeast can become wrinkled and wizened after about 1 month of soaking in fermented liquor (Wang et al., 2018; Kulkarni et al., 2019). The rough surface allows easier access for metal ions for the active sites, facilitating the biosorption process (Zinicovscaia et al., 2023). In the present study, cells were partly aggregating. Particle distribution measurements revealed most particles (45%) were below 100 μm, 29% were between 0.1 and 0.5 mm and approximately 26% of the particles were bigger than 0.5 mm (Supplementary Figure S6).

Following metal biosorption, characteristic peaks for aluminum (1.49 keV), copper (0.93 keV), zinc (1.01 keV) and nickel (0.85 keV) (Socrates, 2001) Klicken oder tippen Sie hier, um Text einzugeben. were observed alongside all other components identified in the untreated biomass (sulfur at 2.31 keV and phosphorous at 2.01 keV) (Figure 2B). The presence of these metal ions on the surface of the biomass after biosorption as observed in the EDS analysis confirms the successful biosorption by spent brewer’s yeast. The distinct peak of potassium at 3.31 keV is only visible for the lyophilized control biomass and disappears once the metal adsorption takes place which points to ion-exchange between potassium and metal ions on the yeast surface as one of the main mechanisms involved in biosorption (Dong et al., 2009).

### 3.4 The effect of physical and chemical pre-treatment methods for yeast biomass

Various pre-treatment methods have been described in literature to optimize the yeast cell surface for biosorption (Yaashikaa et al., 2021). Therefore, the effect of different physical and chemical pre-treatment methods on the biosorption efficiencies were tested. When using the untreated yeast biomass, recovery rates of 45.9% for Cu^2+^, 51.8% for Zn^2+^ and 29.1% for Ni^2+^ were achieved. The physical pre-treatment methods did not improve the biosorption efficiencies for those metals (Figure 1B). For Al^3+^, however, 57.6% of the metal was removed from the solution with the untreated biomass, and 60.2% from the solution with lyophilized biomass. The other 3 pre-treatment methods did not improve the biosorption of Al^3+^, and after sonication of the biomass precipitation was observed. In addition, there was no significant increase in biosorption after chemical pre-treatment with one exception. When the yeast biomass was pre-treated with sodium hydroxide, the biosorption efficiency of Cu^2+^ was significantly increased by 5.7% (Figure 1C). An increase in biosorption efficiency for Cu^2+^ of caustic treated yeast biomass was also described by Göksungur et al. (2005) and could be explained by the removal of proteins from the cell surface that could form non-adsorbable complexes with the metal ions. As observed by Mapolelo and Torto, chemical pre-treatments e.g., with green solvents, disrupt and permeabilize the cell membrane and therefore expose latent metal binding sites (Mapolelo and Torto, 2004). Since the yeast biomass used in these experiments comes from the fermentation industry at the end of the beer brewing process, there is a possibility of natural autolysis resulting in a change of cell morphology and degradation of cell membrane components (Wang et al., 2018) as could be observed in the SEM pictures. These changes in cell surface compared to healthy yeast cells might explain why the pre-treatments did not further improve the biosorption efficiencies.

### 3.5 Selective biosorption from polymetallic solutions

**FIGURE 3:**
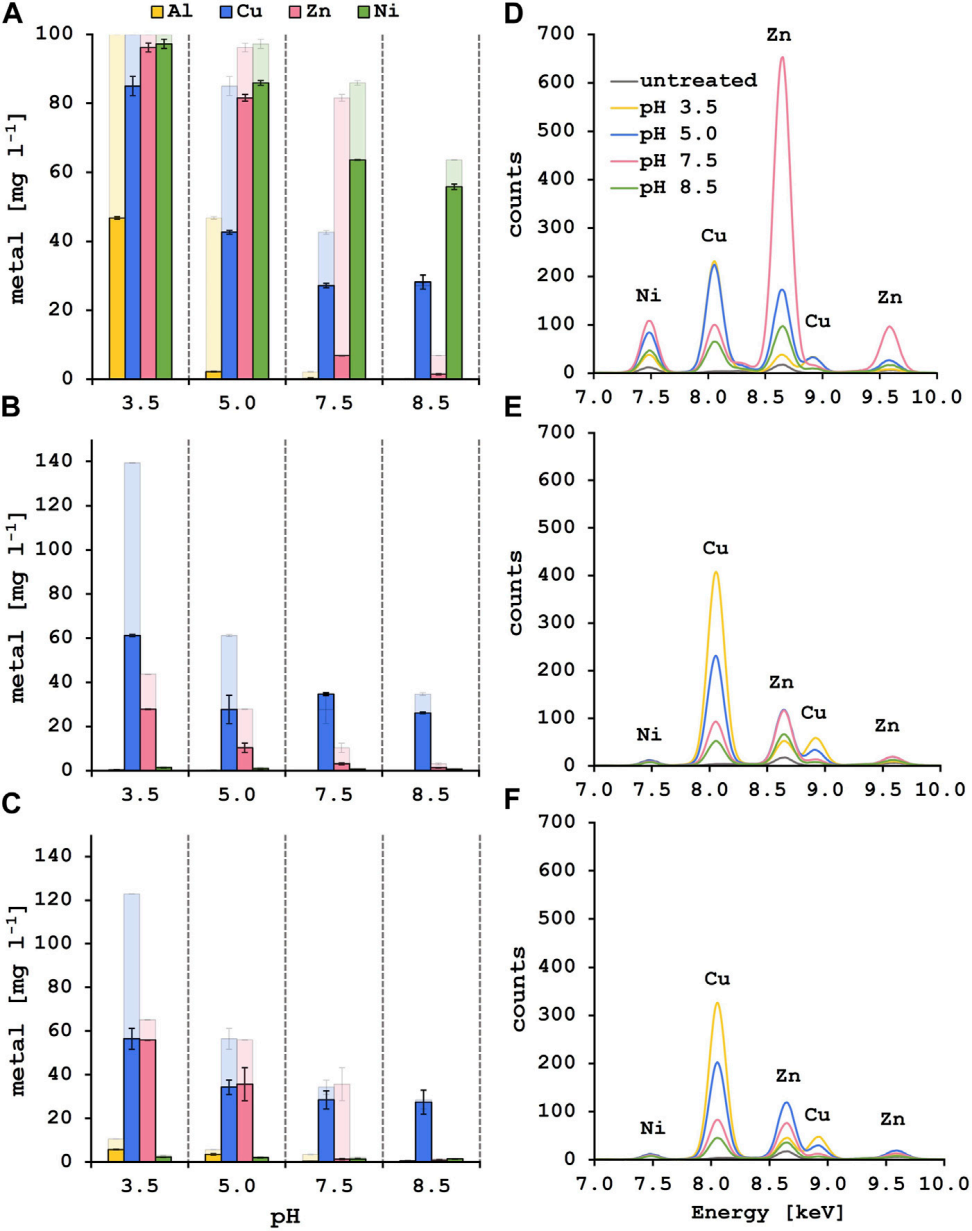
Solution and biomass analysis after the stepwise biosorption process for aluminum, copper, zinc and nickel at the corresponding pH values. Results of the ICP-MS analysis after biosorption from synthetic solution **(A)**, leachate solution 1 **(B)** and leachate solution 2 **(C)**. The strong color indicates the metal concentration [mg l^−1^] after the biosorption step at the indicated pH value, the faded color shows the starting metal concentration [mg l^−1^] of each step. XRF spectra of the yeast biomass after the biosorption from synthetic **(D)**, leachate solution 1 **(E)** and leachate solution 2 **(F)**. The colors indicate at which solution pH the biomass was used. The characteristic peaks for the elements at their specific fluorescent energies are labeled.

Selective biosorption experiments were performed with synthetic solutions containing 100 mg l^−1^ of each metal, adding 10 g l^−1^ of fresh lyophilized yeast biomass after each pH adjustment step. Remaining metal content in the supernatant was analyzed by ICP-MS (Figure 3A). At pH 3.5, 53.2% of Al^3+^ were adsorbed onto the yeast surface whereas most of the Zn^2+^ and Ni^2+^ stayed in solution. In addition, only 15.0% of Cu^2+^ was removed from the solution at the initial biosorption step. Following pH adjustment to 5.0, 42.4% of the remaining Cu^2+^ in solution was adsorbed onto fresh biomass. The residual Al^3+^ was at that point almost completely removed from the solution (>97%). This is explained by the formation of less soluble aluminum hydroxide species starting at pH 4 and hence precipitation, as Costa et al. suggested (Costa et al., 2021). During the next biosorption step at pH 7.5, 74.8% of Zn^2+^ could be adsorbed onto the yeast surface and additional 15.4% of Cu^2+^ were removed. The removal efficiency of Cu^2+^ at this step needs to be evaluated with care as copper starts to precipitate at pH 6.4 (Zinicovscaia et al., 2023). At the last pH step of 8.5, Zn^2+^ was almost completely removed (>98%) whereas more than 50% of the initial Ni^2+^ amount was still in solution. Unexpectedly, the biosorption of Ni^2+^ worked best at pH 7.5 where 23.3% of Ni^2+^ were removed from the solution compared to 7.8% at pH 8.5. While ICP-MS results revealed the remaining metal concentration in the solutions after biosorption, XRF measures the metal appearance on the yeast surface (Figure 3D). Therefore, a similar trend in selective metal adsorption was observed with both methods. A one-way ANOVA analysis was performed to compare the effect of the four different solution pH on the metal recovery efficiencies revealing a significant difference in nickel adsorption between pH 7.5 and 8.5, indicating a more efficient nickel removal at pH 7.5 (Table 3). When measuring the yeast biomass after the adsorption in synthetic solution, there is a characteristic peak for Cu^2+^ visible after the adsorption at pH 3.5 and 5.0 compared to lower peaks at pH 7.5 and 8.5. One-way ANOVA analysis revealed statistically significant differences between pH 5.0 and pH 7.5 or 8.5 for the copper adsorption highlighting the higher copper adsorption at pH 5.0 (Table 3). Even more prominent is the Zn^2+^ peak after the biosorption at pH 7.5. As previously observed, the characteristic peak for Ni^2+^ appears at pH 5.0 and 7.5 and a smaller one is observed at pH 8.5. From these experiments it was concluded that the selective biosorption of metals from a synthetic polymetallic solution worked exceptionally well for Al^3+^, Cu^2+^ and Zn^2+^ at their predefined pH values of 3.5, 5.0 and 7.5, respectively, and the same approach was adapted for the metal recovery out of pregnant PCB bioleaching solutions.

**TABLE 3:** Results of the one-way ANOVA analysis along with the *post hoc* Bonferroni correction for multiple comparisons of XRF results during the synthetic solution biosorption experiment. Values for the standard error (SD) and adjusted *p*-values are reported.

pH		Cu		Zn		Ni	
		SD	*p*-value	SD	*p*-value	SD	*p*-value
3.5	5.0	21.3	1.0	11.3	0.042*	5.3	0.079
	7.5	15.0	0.077	19.6	0.006*	2.1	0.005*
	8.5	15.1	0.049*	4.3	0.032*	2.8	0.479
5.0	3.5	21.3	1.0	11.3	0.042*	5.3	0.079
	7.5	10.9	0.047*	8.9	0.002*	3.5	0.123
	8.5	11.6	0.032*	8.6	0.078	3.9	0.066
7.5	3.5	15.0	0.077	19.6	0.006*	2.1	0.005*
	5.0	10.9	0.047*	8.9	0.002*	3.5	0.123
	8.5	0.714	0.002*	17.5	0.006*	1.1	0.002*
8.5	3.5	15.1	0.049*	4.3	0.032*	2.8	0.479
	5.0	11.6	0.032*	8.6	0.078	3.9	0.066
	7.5	0.714	0.002*	17.5	0.006*	1.2	0.002*

*The mean difference is significant at the 0.05 level.

It is important to note that since the PCB material is very heterogenous there can be other chemical compounds present in the leachate solutions which could interfere with the biosorption capacity for the four metals investigated. Vijayaraghavan and Balasubramanian state that the presence of co-ions strongly affects the removal capacity of the biosorbent towards the ion of interest. Since several chemical groups and components of the biomass surface play an essential role in the passive process of biosorption, complicated interactions are expected in the presence of many ions (Vijayaraghavan and Balasubramanian, 2015). Kulkarni and co-workers for instance described the competitive behavior of Ni^2+^ and Cd^2+^ for vacant sites on brewery sludge (Kulkarni et al., 2019). Moreover, *S. cerevisiae* was investigated by several authors for the removal of other metal ions, e.g., Pb^2+^ (Stathatou et al., 2022), Co^2+^ (Savastru et al., 2022) or Cr^6+^ (De Rossi et al., 2018).

The two different PCB leachate solutions analyzed in this study have very high Cu^2+^ and Zn^2+^ concentrations (Table 2). Both solutions were diluted to reach suitable concentrations of the metals as determined by the DoE screening. There was approximately 75 and 40 times less Ni^2+^ than Cu^2+^ in solution 1 and 2, respectively, and the Al^3+^ content in both leaching solutions was even lower. Their metal recovery rates could therefore not be evaluated. Metal ions such as Cr^6+^, Co^2+^ or Mn^2+^ that could potentially interfere with biosorption were very low in concentration and others like Cd^2+^, Fe^2+^ or Pb^2+^ were under the detection limit for ICP-MS (Supplementary Table S1) and were not considered in further discussion. It is worth mentioning that there was an increase in Mg^2+^ concentration in the synthetic as well as in both leachate solutions measured after each biosorption step (Supplementary Table S1). This observation is in line with the results of several other authors who describe the displacement of cellular metal ions (K^+^, Mg^2+^, Na^+^, Ca^+^) as evidence that ion exchange is the dominant mechanisms in biosorption of heavy metals (Torres, 2020; Zinicovscaia et al., 2020).

Interestingly, after the first biosorption at a pH of 3.5, 56.1% of Cu^2+^ in leachate solution 1% and 54.1% in leachate solution 2 were recovered. In addition, 36.2% of Zn^2+^ in solution 1% and 14.2% in solution 2 were adsorbed. When pH was adjusted to 5.0, an additional 24.0% and 18.0% of Cu^2+^ and 40.1% and 31.0% of Zn^2+^ were removed out of solution 1 and 2. After that the Cu^2+^ concentration in the solutions stayed approximately the same during the last two pH steps. At pH 7.5 almost all the remaining Zn^2+^ was removed from the diluted leachate solutions (>92% total removal of Zn^2+^ for leachate solution 1 and >97% for leachate solution 2) which was the same for the synthetic solution where a total removal of Zn^2+^ of 93.2% was achieved after biosorption at pH 7.5 (Figures 3A–C). The maximum Cu^2+^ removal from the PCB leachate solutions is approximately 80% for leachate solution 1% and 72% for leachate solution 2 at pH 5.0. These results fit well with the biosorption of Cu^2+^ from the synthetic solution where a total of 72.8% of Cu^2+^ was removed at pH 7.5. These observations for the leachate solutions were reinforced by the XRF results where a peak emerged after adsorption at pH 3.5. This characteristic Cu^2+^ peak is also observed at pH 5.0 and still visible at pH 7.5 where the specific peak for Zn^2+^ appeared (Figures 3E,F). This indicates that Al^3+^ ions preferentially bind to the yeast surface at pH 3.5 but in the absence of Al^3+^, Cu^2+^ ions bind to the yeast surface also at a lower pH value. It is described in literature that the sorption capacities of different metal ions follow the order of decreasing electronegativity (Igberase et al., 2017). The electronegativity of aluminum (1.61) is lower than that of copper (1.90) pointing to a higher affinity of the Al^3+^ ions to the yeast surface. Thus, with the absence of Al^3+^ ions in the leachate solutions and the experimental conditions presented here, it would be possible to perform the Cu^2+^ biosorption at pH 3.5 and directly adapt the pH to 7.5 for the zinc biosorption without an additional step at pH 5.0.

### 3.6 Sequential biosorption and desorption studies

**FIGURE 4:**
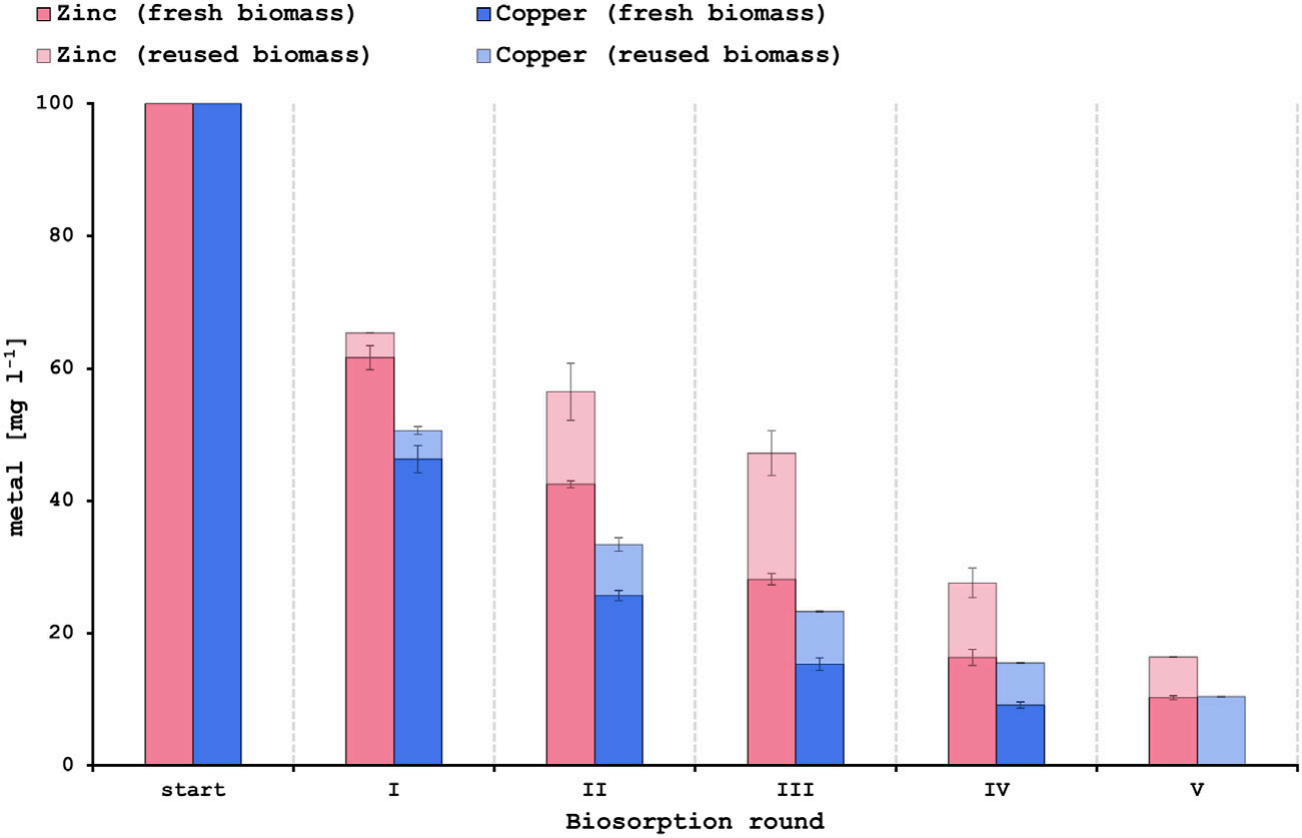
Results of the sequential biosorption experiments using either fresh biomass (strong color) or reused biomass after desorption with biogenic sulfuric acid (fainted color) for each new round.

The capacity for regeneration and reuse of a biosorbent is of high importance since it increases the economic and environmental feasibility of the biosorption process (Costa et al., 2021). Reuse of the yeast biomass could make the metal recovery even more cost-effective while at the same time lowering the dependency on the continuous supply of the adsorbent (Costa et al., 2020). As described in Section 3.5 of this study, the PCB leachate solutions had a high concentration of Cu^2+^ and Zn^2+^ but contained only very low amounts of Al^3+^ and Ni^2+^. Hence, first regeneration and reuse tests were performed for the biosorption of Cu^2+^ and Zn^2+^. Sequential biosorption experiments with synthetic single-metal solutions already showed promising results by increasing the metal removal capacity up to 90% after 4 rounds for Cu^2+^ and after 5 rounds for Zn^2+^ when using fresh biomass for each round (Figure 4). In this study biogenic sulfuric acid that was produced in a previous project (Kremser et al., 2022a) was applied for the desorption of metal ions from the yeast surface. Diluted biogenic sulfuric acid at a pH of 1.2 could be effectively used to desorb Zn^2+^ and Cu^2+^ ions, therefore enabling the reuse of the biosorbent and reaching metal removal efficiencies of up to 83.6% for Zn^2+^ and 89.6% for Cu^2+^ after 5 rounds (Figure 4). However, Cu^2+^ biosorption was more efficient than Zn^2+^ biosorption when reusing the yeast biomass. Studies have shown that sulfuric acid at pH 1.1 can be efficiently used to desorb Cu^2+^ ions (Jung et al., 2022). On the other hand, hydrochloric acid is most commonly used for eluting heavy metal ions requiring different eluent concentrations for the various metals (Yaashikaa et al., 2021). Most biosorbents rely on ion-exchange as a main biosorption mechanism and desorption with mild to strong acids results in exchanging the adsorbed metal ions on the biosorbent surface with protons. Considering that acidic solutions are common waste streams (Vijayaraghavan and Balasubramanian, 2015) this further underlines the merits of using acids as desorbing agents.

## 4 Conclusion

Several studies have investigated the removal of specific metal ions from different waste stream highlighting solution pH as one of the most important parameters for successful metal recovery. In this study, the optimal pH values for aluminum, copper, zinc and nickel biosorption were determined and combined to a stepwise biosorption approach. Using an industrial waste-biomass such as spent brewer’s yeast kept the costs of this process low. Moreover, spent brewer’s yeast proofed to be an excellent candidate for the selective metal recovery from PCB leachate solutions containing high amounts of Cu^2+^ and Zn^2+^. High recovery rates of Al^3+^, Cu^2+^ and Zn^2+^ could be achieved and a preferential binding of Al^3+^ over Cu^2+^ was demonstrated at a low pH value. In addition, it was established that the yeast biomass could be reused several times to increase the removal of Cu^2+^ and Zn^2+^ from synthetic solutions, making the process even more economically feasible. Nevertheless, the parameters for the Ni^2+^ biosorption can still be improved to reach higher recovery rates. The current study showed that a stepwise biosorption approach using spent brewer’s yeast can be used for the selective recycling of metals from PCB leachate solutions. However, further studies focusing on different waste streams need to include a careful analysis of potential interfering metal ions.

## Data Availability

The original contributions presented in the study are included in the article/Supplementary material, further inquiries can be directed to the corresponding author.
