# Supplementary material for: Spent brewer’s yeast as a selective biosorbent for metal recovery from polymetallic waste streams
Source: Front Bioeng Biotechnol. 2024 Mar 12;12:1345112. doi: 10.3389/fbioe.2024.1345112 (PMC10963448; doi:10.3389/fbioe.2024.1345112)
Supplement: Supplementary file 1 [file DataSheet1.docx]

Spent brewer’s yeast as a selective biosorbent for metal recovery from polymetallic waste streams

**Anna Sieber^1^, Leon Robert Jelic^2^, Klemens Kremser^2,3*^, Georg M. Guebitz^2,3^**

^1^K1-MET GmbH, 4020 Linz, Austria

^2^University of Natural Resources and Life Sciences, Vienna, Department of Agrobiotechnology, IFA-Tulln, Institute of Environmental Biotechnology, 3430 Tulln an der Donau, Austria

^3^Austrian Centre of Industrial Biotechnology, 3430 Tulln an der Donau, Austria


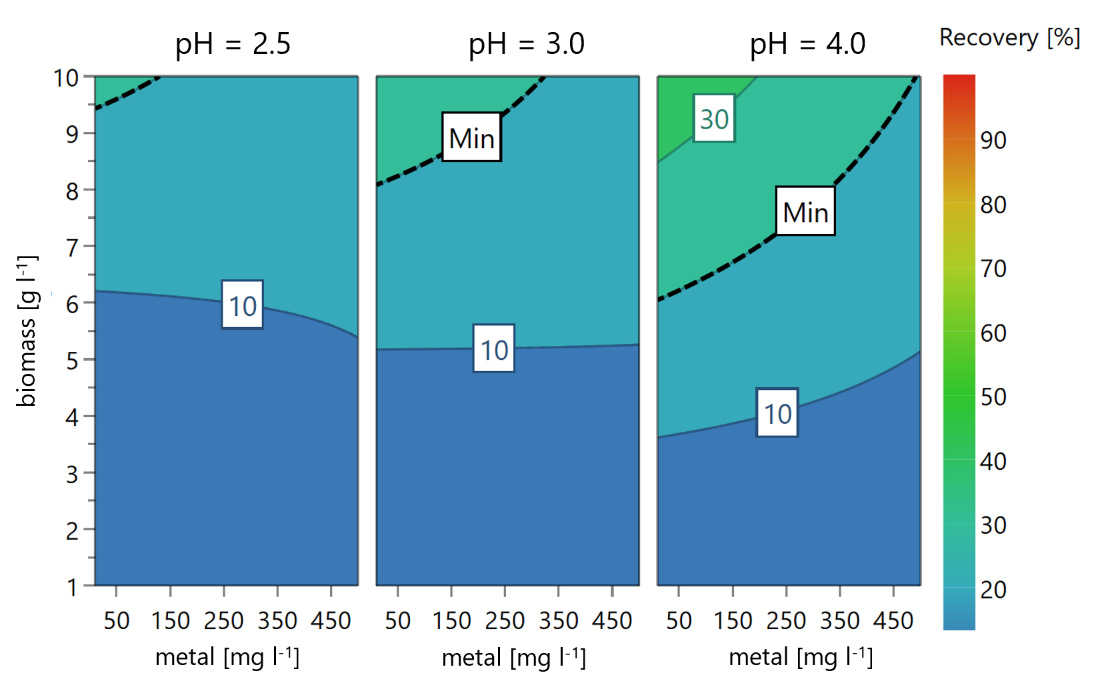


**Supplementary Figure 1**. Contour plot showing the DoE results for the aluminum biosorption at pH 2.5, 3.0 and 4.0. The color gradient symbolizes the recovery rate [%].


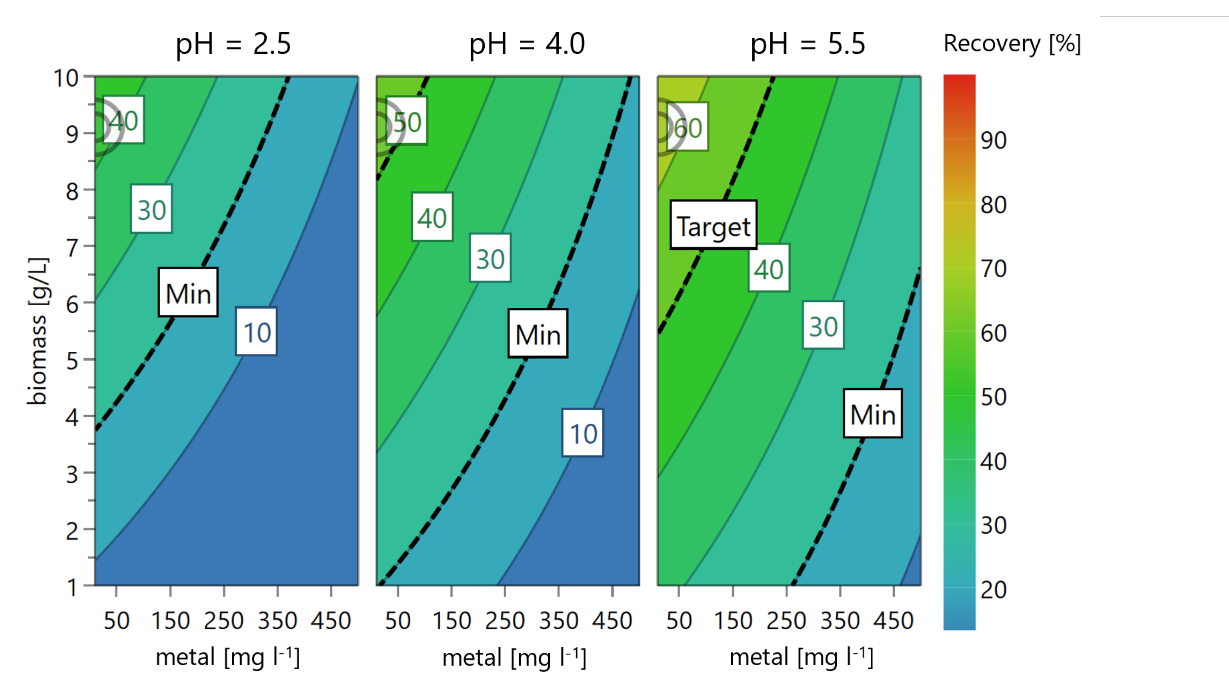


**Supplementary Figure 2.** Contour plot showing the DoE results for the copper biosorption at pH 2.5, 4.0 and 5.5. The color gradient symbolizes the recovery rate [%].


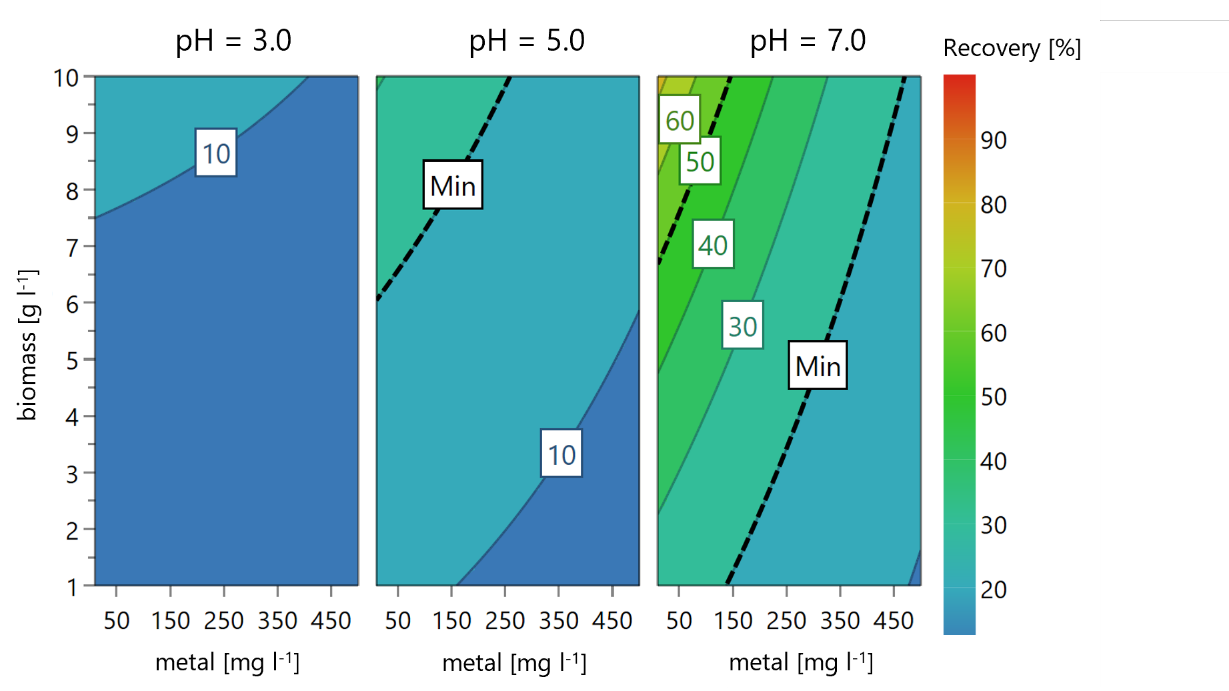


**Supplementary Figure 3**. Contour plot showing the DoE results for the zinc biosorption at pH 3.0, 5.0 and 7.0. The color gradient symbolizes the recovery rate [%].


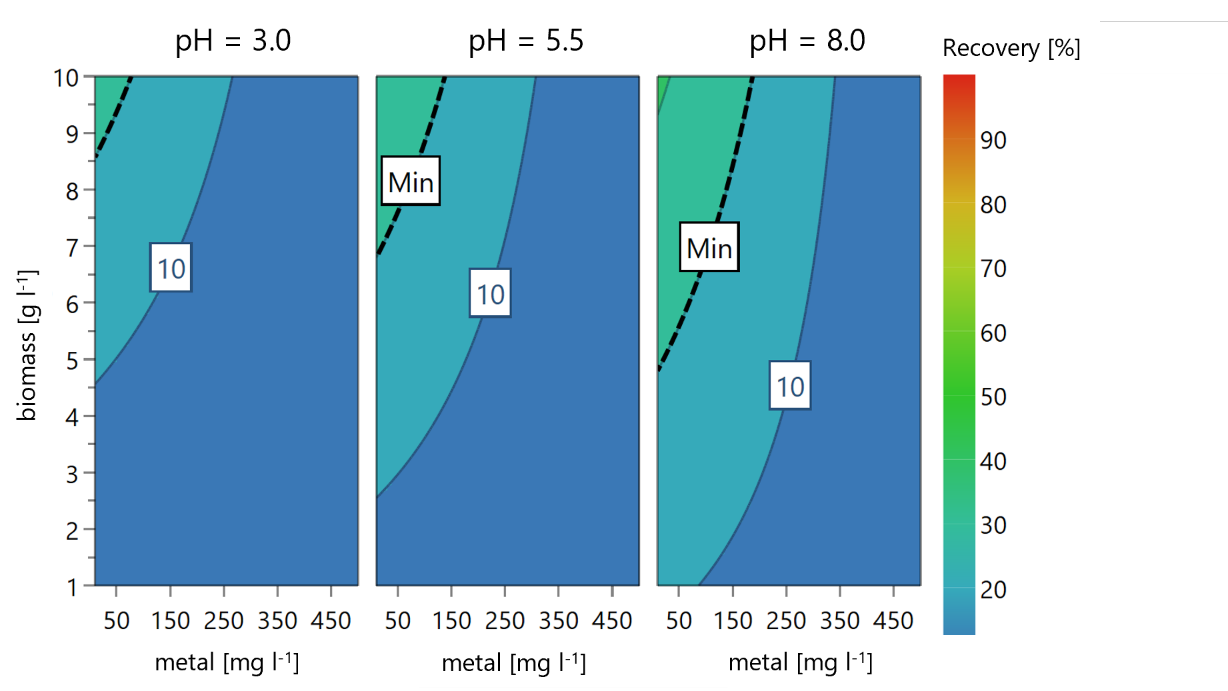


**Supplementary Figure 4**. Contour plot showing the DoE results for the nickel biosorption at pH 3.0, 5.5 and 8.0. The color gradient symbolizes the recovery rate [%].


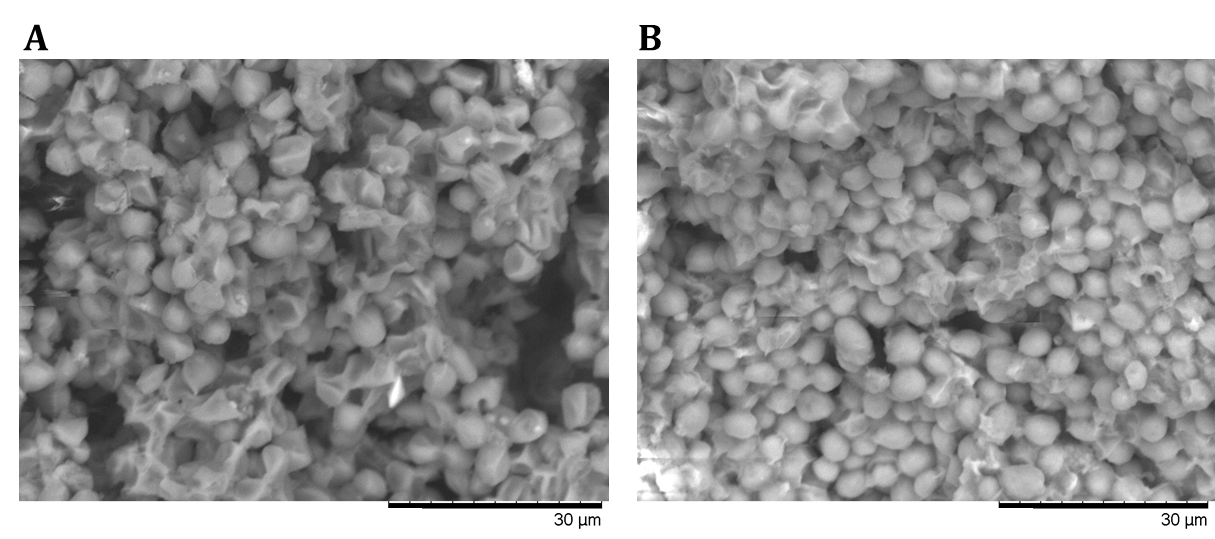


**Supplementary Figure 5**. SEM images at 2000x magnification of lyophilized S. cerevisiae biomass before (A) and after Al^3+^ biosorption (B).


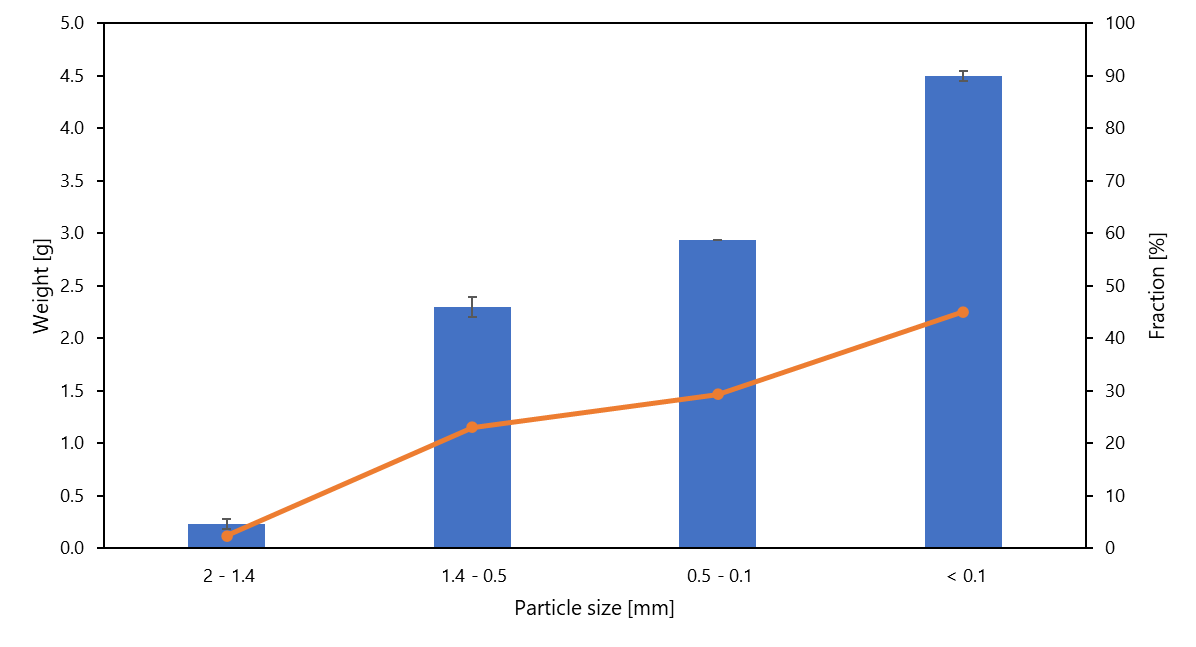


**Supplementary Figure 6.** Particle size distribution of lyophilized yeast biomass obtained by sieving. The blue bars indicate the weight [g] of the particles retained on each sieve (n=3). The orange line shows the percentage [%] of each fraction.

**Supplementary Table 1.** ICP-MS results of different metal ions after the stepwise biosorption process from synthetic and leachate solutions at the corresponding pH values. The values for the concentrations and standard deviation (SD) are depicted in ppm for Mg and ppb for Cr, Mn, Fe, Co, Cd and Pb. The limit of detection (LOD) is reported in the last row for each metal ion.

|  | **pH** | **Mg** | **Cr** | **Mn** | **Fe** | **Co** | **Cd** | **Pb** |
| --- | --- | --- | --- | --- | --- | --- | --- | --- |
|  |  | ppm ± SD | ppb ± SD | ppb ± SD | ppb ± SD | ppb ± SD | ppb ± SD | ppb ± SD |
| **Synthetic solution** | 3.5 | 16.45 ± 0.07 | 6.00 ± 2.10 | 71.45 ± 1.95 | <LOD | 9.20 ± 1.70 | <LOD | <LOD |
|  | 5 | 32.78 ± 0.15 | 3.00 ± 0.00 | 126.10 ± 0.60 | <LOD | 7.95 ± 0.15 | <LOD | <LOD |
|  | 7.5 | 45.97 ± 0.44 | 2.35 ± 0.05 | 121.95 ± 2.25 | <LOD | 6.00 ± 0.20 | <LOD | <LOD |
|  | 8.5 | 57.85 ± 0.43 | 2.90 ± 0.20 | 117.55 ± 0.55 | <LOD | 5.65 ± 0.15 | <LOD | <LOD |
| **Leachate solution 1** | 3.5 | 15.98 ± 0.02 | 18.65 ± 0.25 | 60.75 ± 1.25 | <LOD | 3.05 ± 0.85 | <LOD | <LOD |
|  | 5 | 27.89 ± 0.71 | 10.10 ± 1.80 | 76.35 ± 6.55 | <LOD | 1.90 ± 0.20 | <LOD | <LOD |
|  | 7.5 | 35.20 ± 0.57 | 12.00 ± 0.00 | 69.45 ± 3.45 | <LOD | 1.80 ± 0.00 | <LOD | <LOD |
|  | 8.5 | 41.90 ± 1.27 | 14.55 ± 3.85 | 62.65 ± 6.75 | <LOD | 5.55 ± 3.25 | <LOD | <LOD |
| **Leachate solution 2** | 3.5 | 19.10 ± 0.59 | 59.85 ± 3.25 | 89.7 ± 12.30 | <LOD | 7.45 ± 2.35 | <LOD | <LOD |
|  | 5 | 34.33 ± 0.29 | 42.05 ± 2.55 | 124.75 ± 3.25 | <LOD | 4.85 ± 0.25 | <LOD | <LOD |
|  | 7.5 | 42.22 ± 1.65 | 19.50 ± 1.40 | 79.45 ± 6.05 | <LOD | 2.85 ± 0.65 | <LOD | <LOD |
|  | 8.5 | 54.64 ± 0.34 | 26.35 ± 1.75 | 77.30 ± 2.30 | <LOD | 4.05 ± 0.85 | <LOD | <LOD |
|  | **LOD** | **0.005** | **2** | **0.5** | **355** | **0.2** | **1** | **0.1** |
